# Supplementary material for: Competing itinerant and local spin interactions in kagome metal FeGe
Source: Nat Commun. 2024 Mar 1;15:1918. doi: 10.1038/s41467-023-44190-2 (PMC10907581; doi:10.1038/s41467-023-44190-2)
Supplement: Supplementary file 1 — Supplementary Information [file 41467_2023_44190_MOESM1_ESM.pdf]

Supplementary Information for
Competing itinerant and local spin interactions in kagome metal FeGe

Lebing Chen,[1] Xiaokun Teng,[1] Hengxin Tan,[2] Barry L. Winn,[3] Garrett E. Granorth,[3] Feng Ye,[3] D. H. Yu,[4] R. A. Mole,[4] Bin Gao,[1] Binghai Yan,[2] Ming Yi,[1] and Pengcheng Dai[1]

[1]*Department of Physics and Astronomy, Rice University, Houston, Texas 77005, USA*
[2]*Department of Condensed Matter Physics, Weizmann Institute of Science, Rehovot 7610001, Israel*
[3]*Neutron Scattering Division, Oak Ridge National Laboratory, Oak Ridge, Tennessee 37831, USA*
[4]*Australian Nuclear Science and Technology Organisation, Lucas Heights, New South Wales 2234, Australia*

Supplementary Figure 1: Sample characterizations, and the potential CDW structure and its effect on magnetism.

Supplementary Figure 2: Additional data on magnetic excitations in FeGe.

Supplementary Figure 3: Temperature dependence of magnetic Bragg peaks in FeGe under 11-T in-plane field.

Supplementary Figure 4: Inelastic neutron scattering data under 2-T in-plane field.

Supplementary Figure 5: Inelastic neutron scattering data under an extended temperature range.

Supplementary Figure 6: Band structure comparison between the FM and AFM phases without spin-orbit-coupling.

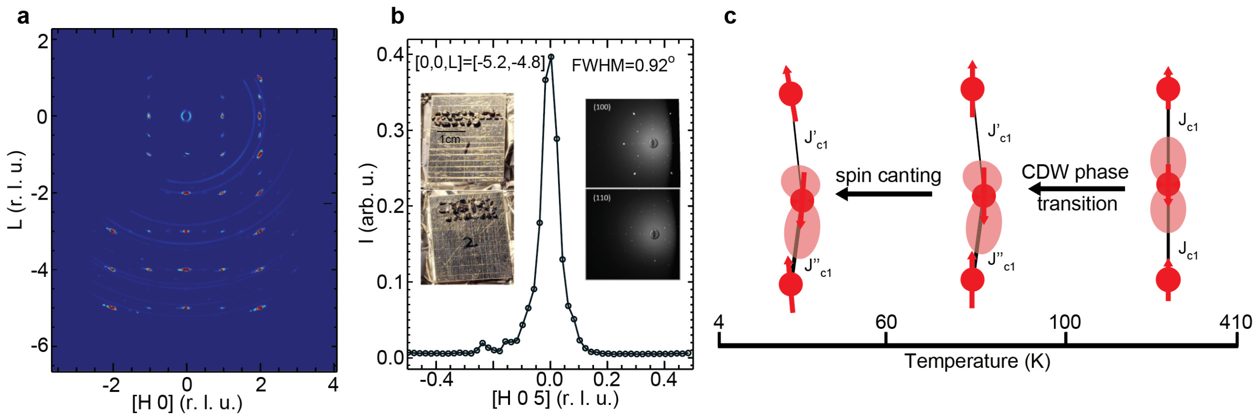

FIG. 1. **Sample characterizations, and the potential CDW structure and its effect on magnetism.** (a) Lattice and magnetic Bragg peak of the FeGe sample at T=120K, data taken from ARCS with E_i=45meV. (b) The [H 0 5] Bragg peak, showing the sample mosaic with FWHM=0.92°. The insets show the image of the co-aligned sample and representative Laue patterns of the sample. (c) Exaggerated schematics on the effect of CDW phase transition on the Fe atoms (red solid circles). The modulation of the atom positions can introduce asymmetric anisotropies (red-shaded areas in the center Fe atom), and add inhomogeneity to interlayer exchanges J_c's (black lines).

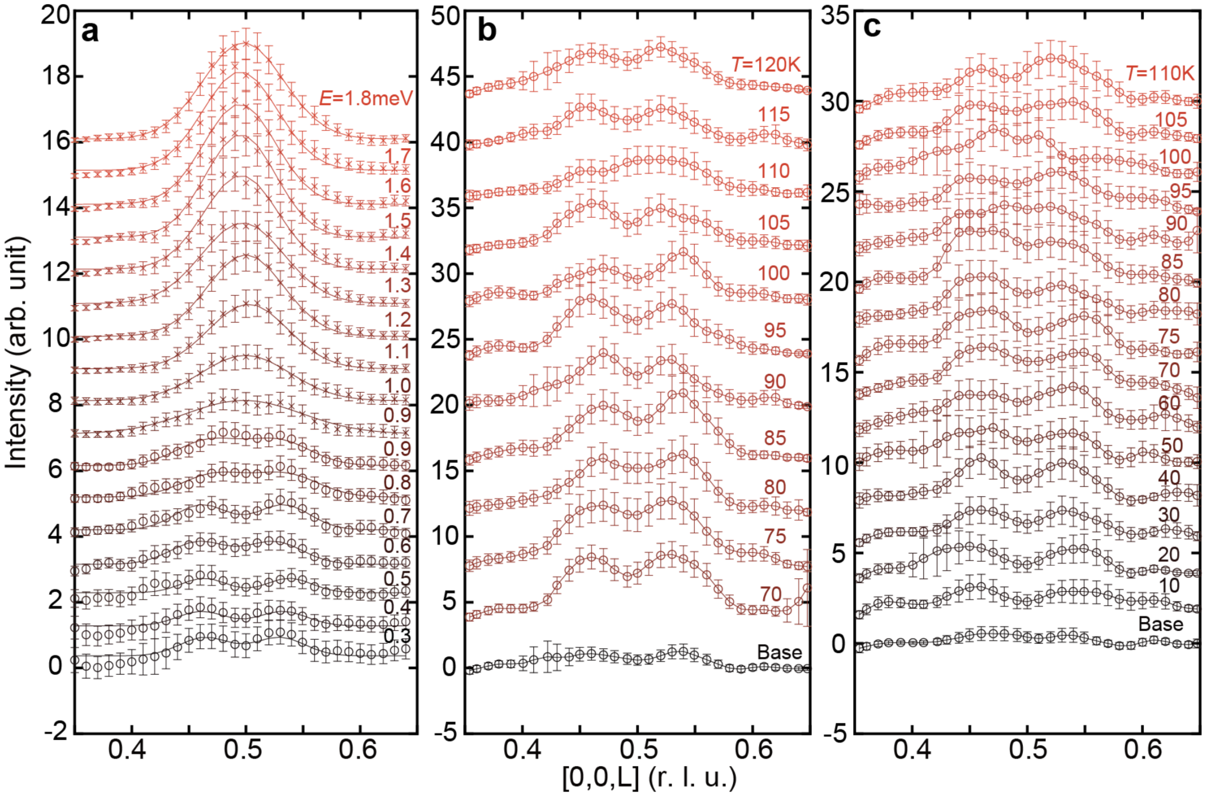

FIG. 2. **Additional data on magnetic excitations in FeGe** (a) The data used for fitting in Fig. 2f of the main text. The circle dots and crosses indicate double- and single-peak fitting, respectively. (b,c) The data used for generating the (b) 0-T and (c) 11-T portions in Fig. 4f. The data points associated with the HYSPEC experiment in Fig. 4f come from integrating the data points here.

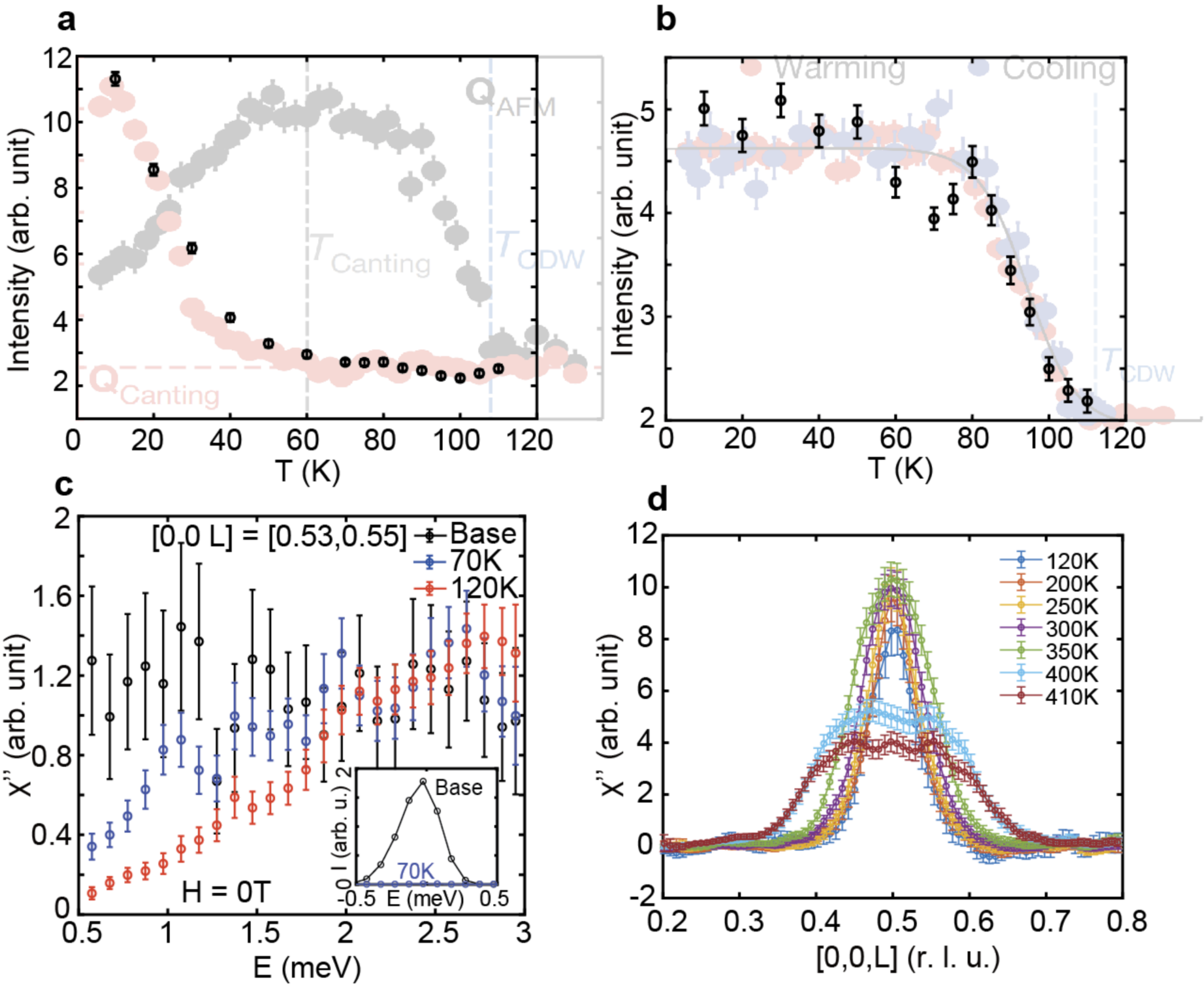

FIG. 3. **Temperature dependence of magnetic Bragg peaks in FeGe under 11-T in-plane field.** (a) T-dependence of the incommensurate Bragg peak, data integrated from $L = [0.52, 0.7]$. (b) T-dependence of the $(0, 0, 0.5)$ Bragg peak, data integrated from $L = [0.48, 0.52]$. The shaded overlays are 0-T data taken from ref.[**?**]. (c) Energy dependence of the spin dynamic susceptibility χ'' under base, 70 K and 120 K and 0 T at q_{IC} . The inset shows the temperature dependence of the incommensurate Bragg peak intensity between base and 70 K. Data in (a-c) are taken at the HYSPEC spectrometer. (d) Temperature dependence of the spin susceptibility χ'' above 120 K at $E = 1.5$ meV. Data taken at the Pelican spectrometer.

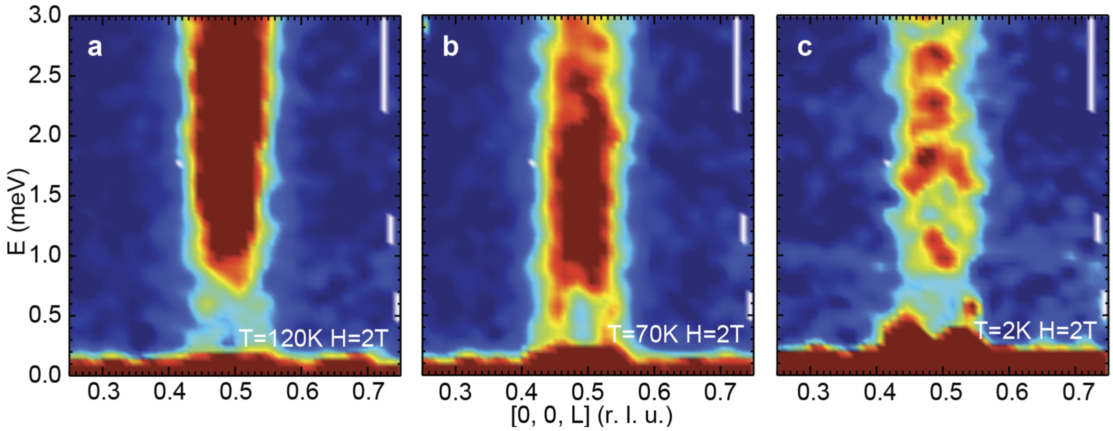

FIG. 4. **Inelastic neutron scattering data under 2-T in-plane field.** Data taken at the HYSPEC spectrometer, under (a) 120 K, (b) 70 K, and (c) 2 K under 2-T in-plane magnetic field.

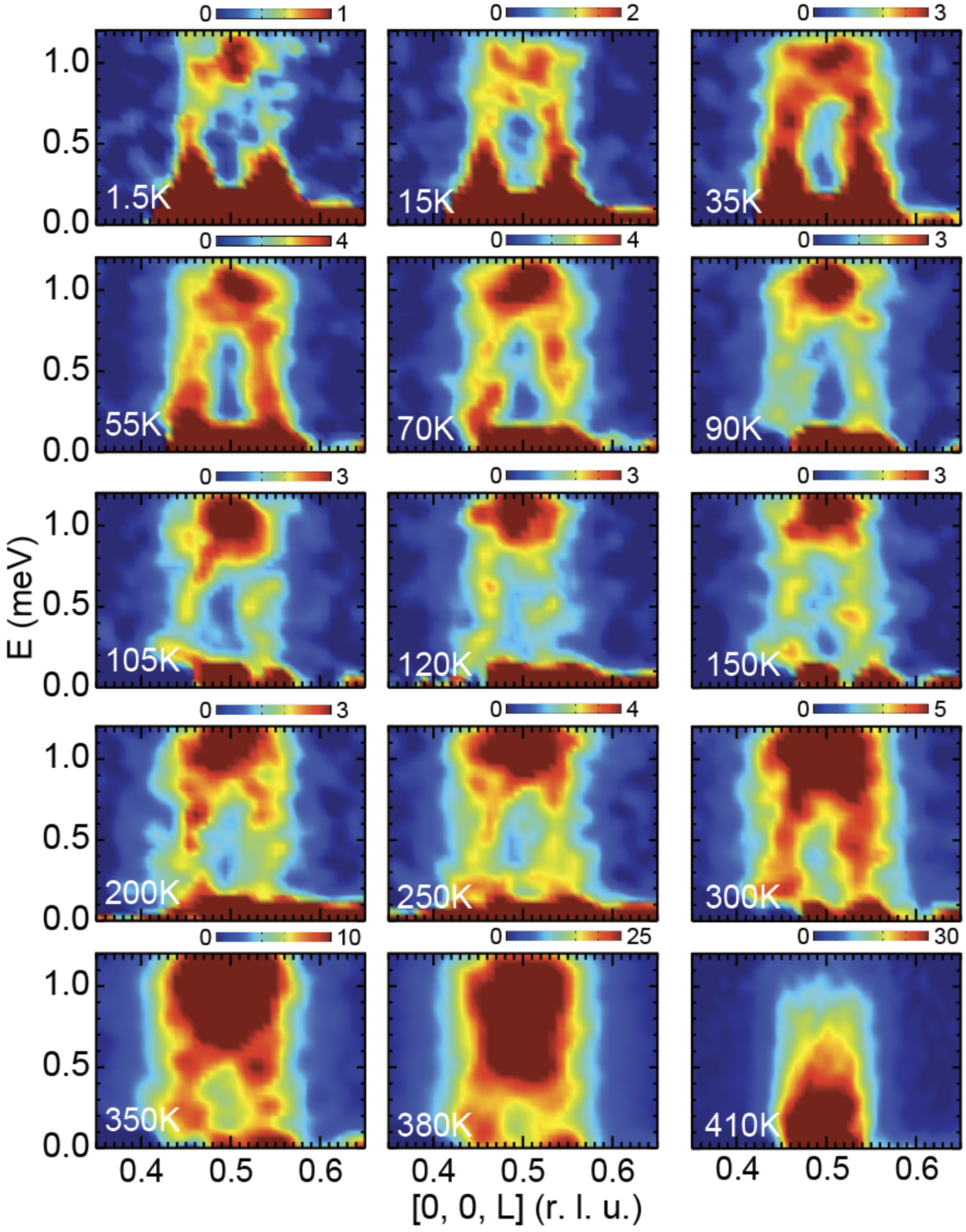

FIG. 5. **Inelastic neutron scattering data under an extended temperature range.** Data taken at the Pelican spectrometer. The same data is used to generate Fig.4e and 4f.

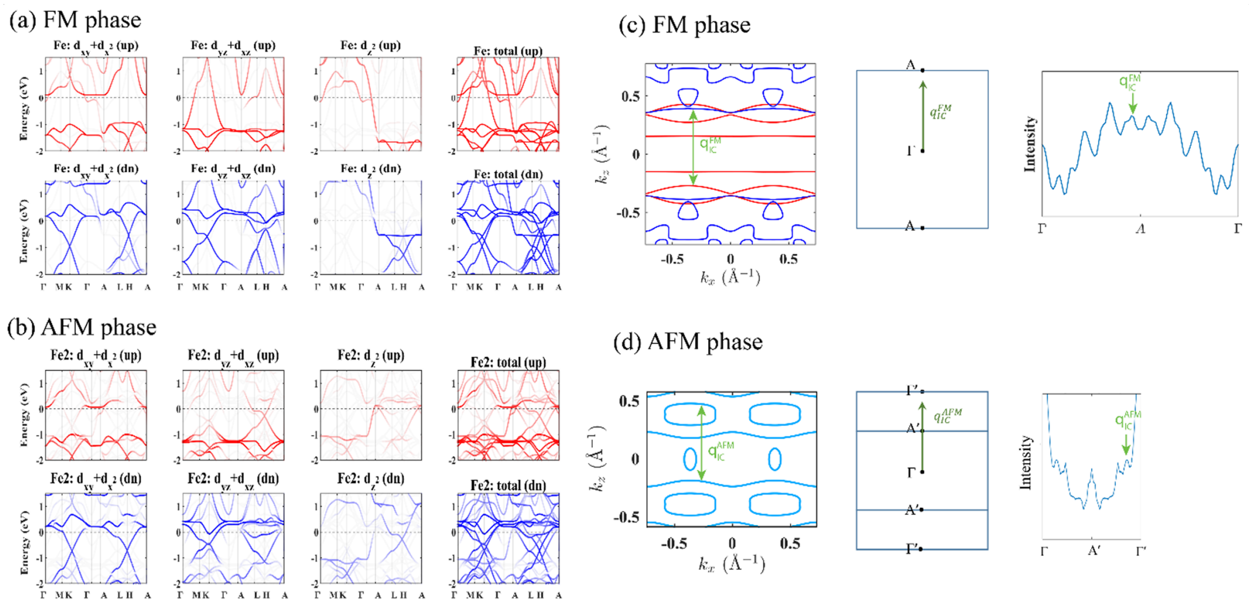

FIG. 6. **Band structure comparison between the FM and AFM phases without SOC.** (a) Spin and orbital resolved band structure of the FM phase. Up and dn in the titles stand for the spin up and down, respectively. (b) Similar to (a) but for the AFM phase. Notice that in (b), the projection is made for one of the two kagome sub-lattices in the AFM phase. (c) The left panel shows the Fermi surfaces of the FM phase in the k_x-k_z plane indicated in Fig.1c in the main text. The red and blue curves are for the spin up and down, respectively. The middle panel shows the nesting vector q_{IC}^{FM} (see the left panel) in the Brillouin zone along the kz direction. The right panel shows the Fermi surface nesting along the z-direction between the spin-up and down Fermi surfaces shown in the left panel. The y-axis ('Intensity') is proportional to the nesting susceptibility $\chi(\boldsymbol{q})$. The nesting vector q_{IC}^{FM} (same as the q_{IC} in Fig.1i in the main text) is indicated. (d) Similar to (c) but for the AFM phase. Notice that the spin-up and spin-down channels degenerate in the AFM phase. In the middle panel, the Brillouin zone folding folds the A point of the FM Brillouin zone to the Γ (0,0,1) of the AFM Brillouin zone; thus, the nesting vector (q_{IC}^{AFM}) in the AFM phase appears close to Γ (see the comparison of the nesting vector in the FM and AFM Brillouin zones in the middle panels of (c) and (d)).
